# Successful Use of Intravenous Immunoglobulin G to Treat Refractory Heparin-Induced Thrombocytopenia With Thrombosis Complicating Peripheral Blood Stem Cell Harvest

**DOI:** 10.1177/2324709618755414

**Published:** 2018-01-29

**Authors:** Devon S. McKenzie, Josephine Anuforo, Jennah Morgan, Elvira Neculiseanu

**Affiliations:** 1 SUNY Downstate Medical Center, New York, NY, USA; 2 St John’s Episcopal Hospital, Far Rockaway, NY, USA

**Keywords:** heparin-induced thrombocytopenia, IV immunoglobulin, refractory heparin-induced thrombocytopenia with thrombosis

## Abstract

Heparin-induced thrombocytopenia is a well-known, life-threatening complication that occurs in 5% of patients exposed to heparin. It causes thrombocytopenia in roughly 85% to 90% of affected individuals, with expected recovery in approximately 4 to 10 days following heparin withdrawal. However, there is an entity known as refractory heparin-induced thrombocytopenia with thrombosis in which patients have prolonged thrombocytopenia, refractory to the current standard of care. We present one such case of a 48-year-old male with R-ISS (Revised International Staging System) stage II kappa light chain multiple myeloma in stringent complete response status postinduction therapy. He developed heparin-induced thrombocytopenia with thrombosis during peripheral blood stem cell harvesting, manifesting as acute right coronary artery thrombus and severe thrombocytopenia. Although his clinical course was prolonged, he was ultimately successfully treated with intravenous immunoglobulin G 500 mg/kg/day over 4 days.

## Introduction

Refractory heparin-induced thrombocytopenia with thrombosis (HITT) is a rarely reported manifestation where the immunoglobulin G (IgG) antibodies against endogenous platelet factor 4 (PF4) in complex with heparin and resultant thrombocytopenia persist beyond the expected time to resolution (~4-10 days) after initiation of conventional therapy. There are currently no specific guidelines as to the management of HITT refractory to standard treatment. However, there is evidence showing efficacy and durable responses with the use of intravenous immunoglobulin G (IVIgG). This case aims to corroborate that evidence; it also suggests that patients do not necessarily need to achieve a normal platelet count to be bridged to warfarin if they are adequately anticoagulated with a seroconverted serotonin release assay (SRA).

## Case Presentation

We present the case of a 48-year-old African American male with a history R-ISS (Revised International Staging System) stage II kappa light chain multiple myeloma in stringent complete response, postinduction therapy. He was admitted for ST-elevation myocardial infarction3 days after completing peripheral blood stem cell harvest and was subsequently found to have refractory HITT.

He originally presented to an outside facility 1 year prior to presentation at our institution, with severe 10/10 pain in his right lower back and hip associated with a 20 lbs weight loss. His laboratory findings at that time showed calcium of 8.5 mg/dL, serum creatinine of 1.4 mg/dL (attributed to hypertensive nephropathy worsened by newly diagnosed multiple myeloma), and lactate dehydrogenase peak of 703 U/L (313-618 U/L). Serum electrophoresis showed no M spike. Free light chain (FLC) ratio was 240.3. Skeletal survey showed a large lytic lesion in the posterior right iliac crest. Bone marrow biopsy sample was inadequate with near absence of hematopoietic marrow. However, the absence of an M spike, the FLC >100, and lytic lesion on skeletal survey were sufficient to make the diagnosis of FLC myeloma.

He was started on dexamethasone and underwent palliative radiation to the right iliac crest. He was subsequently treated with 6 cycles of cyclophosphamide, bortezomib, and dexamethasone (CyBorD) induction therapy. Postchemotherapy follow-up and repeat bone marrow biopsy panel showed that he was in stringent complete remission with a FLC ratio of 1.39 and total absence of clonal plasma cells on postinduction bone marrow biopsy. Hence, he was started on maintenance therapy with bortezomib and dexamethasone, and then referred for stem cell harvest for possible autologous transplant.

**Figure 1.**
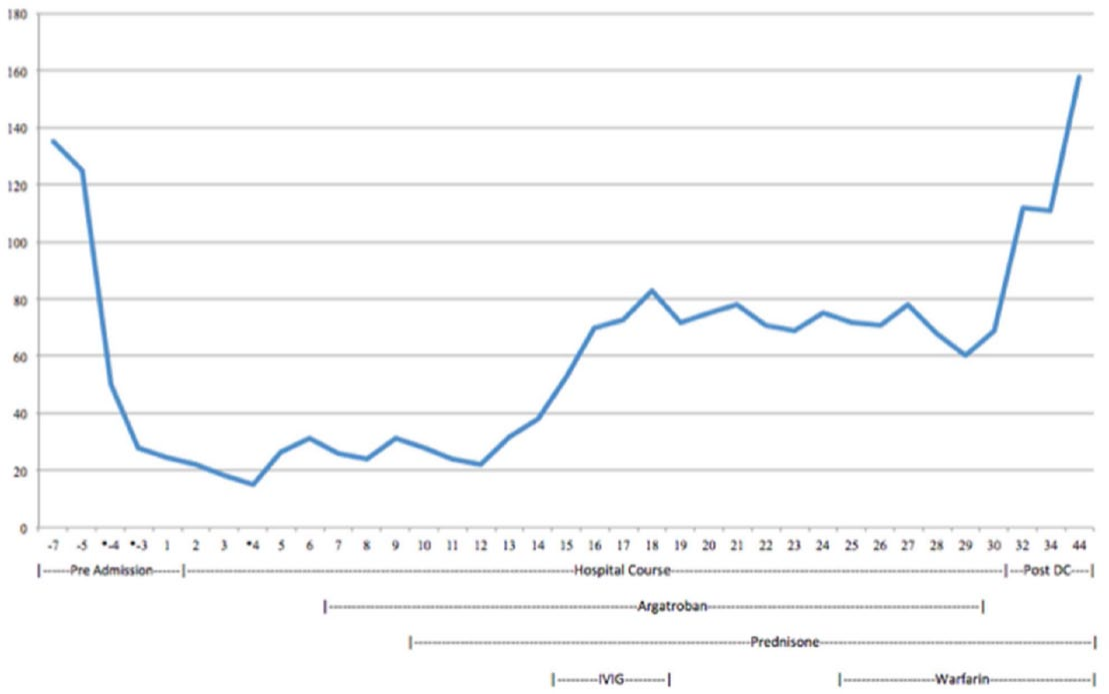
Graph showing platelet trend. The graph reflects the patient’s measured platelet count prior to admission, during hospital course, and postdischarge follow-up. Values with an asterisk reflect when the patient received a platelet transfusion.

The peripheral blood stem cell harvesting using plerixafor and neupogen was started 1 week prior to admission to our facility. Over 3 million cells were collected, but further harvesting was held due to progressive thrombocytopenia to a nadir of 28 000/µL. This was thought to be a result of platelet loss during apheresis (Figure 1) for which he was given 1 unit of single donor platelet on day 3 of stem cell harvesting.

**Figure 2.**
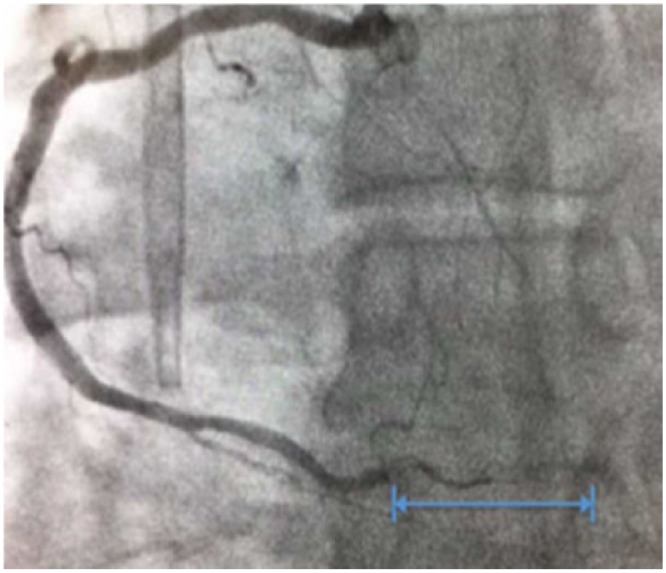
The image shows the occluded portion of the distal segment of the right coronary artery obtained during cardiac catheterization on day 1 of admission. The blue marking highlights the visibly occluded portion of the artery.

He presented to our facility with acute, nonradiating, nonpleuritic left-sided chest pain with ST segment elevation in the inferior leads on the electrocardiogram and troponin peak of 34.66 ng/mL. He was diagnosed with ST-elevation myocardial infarction and loaded with heparin, plavix, and aspirin. He had subsequent cardiac catheterization, which discovered a distal right coronary artery (RCA) thrombus not amenable to stent placement (Figure 2); hence, only a balloon angioplasty was done.

Postprocedure physical examination was overall unremarkable with no evidence of bleeding, hepatosplenomegaly, and no signs or symptoms suspicious for deep vein thrombosis/venous thromboembolism. His laboratory findings on day 1 of admission were as follows: white blood cell count of 28.18 cells/µL, hemoglobin of 17.5 g/dL, and platelet count of 26 000/µL. Peripheral smear showed no abnormalities other than a decreased number of platelets per high-powered field. Heparin-induced thrombocytopenia enzyme-linked immunosorbent assay (HIT ELISA), heparin-induced platelet aggregation assay, and SRA were sent on day 3 after platelet count continued trending down to 21 000/µL, reaching a nadir at 14 000/µL on day 5. His thrombocytopenia was believed to be secondary to apheresis (as we did not have medical records from his previous hospital) and he was given 1 unit of platelet on day 5, which increased his platelet count from 14 000/µL to 31 000/µL. HIT ELISA results came back on day 6 as 2.824 OD (optical density) with a positive low-dose heparin SRA (61%). This confirmed the diagnosis of HITT; hence, he was started on argatroban drip and subcutaneous heparin for deep venous thrombosis prophylaxis was stopped. After 7 days of receiving argatroban and being off all heparin products, there was no significant response in his platelet count, which was 26 000/µL on day 6 and 22 000/µL after day 13.

Additionally, as he had no physical examinations or laboratory findings suggestive of alternative causes of thrombocytopenia. He was also started on 1 mg/kg/day prednisone on day 9 for possible immune thrombocytopenic purpura with no improvement. Given that his platelet count failed to improve in the absence of heparin, and there are recently published data supporting the utility of IVIG in refractory HITT, he was started on IVIgG 500 mg/kg/day from days 14 to 18 of hospitalization. This increased his platelet count from 21 000/µL on day 14 to 70 000/µL on day 18. Thereafter, it fluctuated between 68 000/µL and 83 000/µL.

Although he did not achieve the recommended platelet goal of 150 000/µL, his repeat low-dose heparin SRA on day 20 showed seroconversion with a decrease from 61% to 10%; hence, he was bridged to warfarin from days 25 to 29 and discharged home with close outpatient follow-up. On follow-up 3 days postdischarge, his platelet count was 112 000/µL with no acute symptoms. He was continued on warfarin and his platelet count ultimately corrected to 158 000/µL, 13 days postdischarge (45 days after his original presentation).

## Discussion

HIT is a well-known, life-threatening complication that occurs in roughly 5% of patients exposed to heparin. The underlying pathophysiology depends on the formation of autoantibodies directed against endogenous PF4-heparin complexes.^1,2^ This results in the coating of the platelets by IgG, which are subsequently removed by macrophages in the reticuloendothelial system, leading to thrombocytopenia in roughly 85% to 90% of affected patients.^3,4^ It also leads to the activation of the platelets by an unknown mechanism, which increases the risk of both arterial and venous thrombosis, occurring in roughly 50% of affected individuals. This phenomenon contributes to consumptive thrombocytopenia.^4^ Once HIT is suspected, the 4T score can be used to determine the pretest probability, with a presumptive diagnosis of HIT if the score shows intermediate or high probability (4T score > 4-8).^4-7^ However, it dictates further confirmatory tests with HIT immunoassays/HIT ELISA, heparin-induced platelet aggregation test, and SRA.

The former is reported in OD and an OD >2 reflects an >89% likelihood of HIT.^8^ The most common site of venous thrombosis is in the lower extremities, but it can also occur in the cardiac vessels and skin. Arterial thrombosis has been reported in the heart, internal organs, and limbs.^9-11^

In our patient, we believe the heparin he received during the peripheral blood stem cell harvesting was the inciting factor for the development of HIT, which manifested as a distal RCA myocardial infarction. At presentation, we did not have access to his outside records, which subsequently showed a clear decrease in his platelet count. In retrospect, his 4T score was 7, indicative of a high probability for HIT (2 points for platelet count fall >50%, 2 points for clear onset between 5 and 10 days of heparin exposure, 2 points for RCA thrombosis, and 1 point for no other possible causes of thrombocytopenia). Hence, he should have been started on therapy at that time. We did send the immunoassays, which confirmed HIT and subsequently replaced all heparin products with a non–heparin anticoagulant (a direct thrombin inhibitor argatroban).^12^ This was done because even in the absence of heparin, HIT patients are at significantly increased risk for thrombosis as there is still continuous platelet activation.^6^ Based on the current recommendations by the American College of Chest Physician, treatment with argatroban should be continued until the platelet count has substantially recovered to above 150 000/µL. Once that target is achieved, the patient should be bridged with a 5-day overlap to a vitamin K antagonist to be continued for at least 3 months.^13^

The expected resolution of thrombocytopenia following the withdrawal of heparin typically occurs in 4 to 10 days^14^; unfortunately, our patient had refractory HITT, which worsened on argatroban-only therapy. Given that other causes of thrombocytopenia were ruled out (refer to case report), he was started on prednisone for possible immune thrombocytopenic purpura with no significant improvement. This only confirmed refractory HIT with thrombosis (HITT).

Review of the literature showed a possible option in IVIgG for treating HITT refractory to standard therapy. It is hypothesized that IVIgG binds to the Fc domain of the IgG targeted against the PF4-heparin complex; consequently, inhibiting antibody-mediated platelet activation. This prevents further platelet destruction with resultant increase in platelet count.^13-19^

There are approximately 13 cases reported in the literature of refractory HIT/HITT effectively treated with IVIgG^18,19^ since 1989. However, the current treatment guidelines do not recommend IVIgG for refractory HITT. Important to note, not all the above-mentioned cases achieved a platelet count >150 000/µL prior to bridging to warfarin, suggesting that a stable improved platelet count or a seroconverted SRA may be substituted for a specific goal for platelet count.

This response was reflected in our patient with an approximate tripling of his platelet count. Although he did not get to 150 000/µL (maximum platelet count prior to bridging was 83 000/µL), he had seroconversion of his SRA (68% to 10%) and was successfully bridged to warfarin with no adverse effects. Unlike the other reported cases in which 1 to 2 g/kg/day of IVIgG was used over 2 to 3 days, we opted for 0.5 mg/kg/day over 4 days. The reason for this lower dosing bordered on the numerous case reports of myocardial infarctions associated with IVIgG use^20-21^ therefore, we erred on the side of caution. His platelet count has since normalized and the patient is doing well.

The essence of this case is that it adds to evidence-based armamentarium supporting the use of IVIgG as an effective agent in patients with severe refractory HIT. It also shows that these patients do not necessarily need to achieve a normal platelet count to be bridged to warfarin if adequately anticoagulated with a seroconverted SRA.

## References

[1] Khandelwal S Arepally GM. Immune pathogenesis of heparin-induced thrombocytopenia. Thromb Haemost. 2017;116:792-798. 10.1160/TH16-01-0074 PMC6260829 27465274

[2] Rauova L Zhai L Kowalska MA Arepally GM Cines DB Poncz M. Role of platelet surface PF4 antigenic complexes in heparin-induced thrombocytopenia pathogenesis: diagnostic and therapeutic implications. Blood. 2006;107:2346-2353. 16304054 10.1182/blood-2005-08-3122 PMC1895727

[3] Linkins LA Dans AL Moores LK et al Treatment and prevention of heparin-induced thrombocytopenia: antithrombotic therapy and prevention of thrombosis, 9th ed: American College of Chest Physicians evidence-based clinical practice guidelines. Chest. 2012;141(2 suppl):495S-e530S. 10.1378/chest.11-2303 PMC3278058 22315270

[4] Warkentin TE Anderson JAM How I treat patients with a history of heparin-induced thrombocytopenia. Blood. 2016;128:348-359. doi:10.1182/blood-2016-01-635003. 27114458

[5] Linkins LA. Heparin induced thrombocytopenia. BMJ. 2015;350:g7566. 25569604 10.1136/bmj.g7566

[6] Greinacher A. Clinical practice. Heparin-induced thrombocytopenia. N Engl J Med. 2015;373:252-261. 26176382 10.1056/NEJMcp1411910

[7] Warkentin TE. Clinical picture of heparin-induced thrombocytopenia (HIT) and its differentiation from non-HIT thrombocytopenia. Thromb Haemost. 2016;116:813-822. 27656712 10.1160/TH16-06-0435

[8] Baroletti S Hurwitz S Conti NA Fanikos J Piazza G Goldhaber SZ. Thrombosis in suspected heparin-induced thrombocytopenia occurs more often with high antibody levels. Am J Med. 2012;125:44-49. 22075045 10.1016/j.amjmed.2011.06.025

[9] Giossi A Del Zotto E Volonghi I et al Thromboembolic complications of heparin-induced thrombocytopenia. Blood Coagul Fibrinolysis. 2012;23:559-562. 22688553 10.1097/MBC.0b013e3283502989

[10] Ayala E Rosado MF Morgensztern D Kharfan-Dabaja MA Byrnes JJ. Heparin-induced thrombocytopenia presenting with thrombosis of multiple saphenous vein grafts and myocardial infarction. Am J Hematol. 2004;76:383-385. doi:10.1002/ajh.20114. 15282674

[11] LaMonte MP Brown PM Hursting MJ. Stroke in patients with heparin-induced thrombocytopenia and the effect of argatroban therapy. Crit Care Med. 2004;32:976-980. 15071388 10.1097/01.ccm.0000119426.34340.e2

[12] Hong AP Cook DJ Sigouin CS Warkentin TE. Central venous catheters and upper-extremity deep-vein thrombosis complicating immune heparin-induced thrombocytopenia. Blood. 2003;101:3049-3051. 12506031 10.1182/blood-2002-05-1448

[13] Lewis BE Wallis DE Hursting MJ Levine RL Leya F. Effects of argatroban therapy, demographic variables, and platelet count on thrombotic risks in heparin-induced thrombocytopenia. Chest. 2006;129:1407-1416. 16778256 10.1378/chest.129.6.1407

[14] Ahmed I Majeed A Powell R. Heparin induced thrombocytopenia: diagnosis and management update. Postgrad Med J. 2007;83:575-582. doi:10.1136/pgmj.2007.059188. 17823223 PMC2600013

[15] Padmanabhan A Jones CG Pechauer SM et al IVIg for treatment of severe refractory heparin-induced thrombocytopenia. Chest. 2017;152:478-485. doi:10.1016/j.chest.2017.03.050. 28427966 PMC5812774

[16] Doucette K DeStefano CB Jain NA Cruz AL Malkovska V Fitzpatrick K. Treatment of refractory onset-heparin-induced thrombocytopenia after thoracic endovascular aortic repair with intravenous immunoglobulin (IVIG). Res Pract Thromb Haemost. 2017;1:134-137. doi:10.1002/rth2.12009. PMC6058197 30046682

[17] Lei BZ Shatzel JJ Sendowski M. Rapid and durable response to intravenous immunoglobulin in delayed heparin-induced thrombocytopenia: a case report. Transfusion. 2017;57:919-923. doi:10.1111/trf.13960. 27943368

[18] Tvito A Bakchoul T Rowe JM Greinacher A Ganzel C. Severe and persistent heparin-induced thrombocytopenia despite fondaparinux treatment. Am J Hematol. 2015;90:675-678. doi:10.1002/ajh.23971. 25683147

[19] Stenton SB Dalen D Wilbur K. Myocardial infarction associated with intravenous immune globulin. Ann Pharmacother. 2005;39:2114-2118. 16288078 10.1345/aph.1G104

[20] Mizrahi M Adar T Orenbuch-Harroch E Elitzur Y. Non-ST elevation myocardial infarction after high dose intravenous immunoglobulin infusion. Case Rep Med. 2009;2009:861370. doi:10.1155/2009/861370. 20182639 PMC2825772

[21] Elkayama O Paran D Milo R et al Acute myocardial infarction associated with high dose intravenous immunoglobulin infusion for autoimmune disorders. A study of four cases. Ann Rheum Dis. 2000;59:77-80. 10627434 10.1136/ard.59.1.77 PMC1752991

